# Supplementary material for: Acetaldehyde makes a distinct mutation signature in single-stranded DNA
Source: Nucleic Acids Res. 2022 Jul 1;50(13):7451–64. doi: 10.1093/nar/gkac570 (PMC9303387; doi:10.1093/nar/gkac570)
Supplement: gkac570_Supplemental_Files [file gkac570_supplemental_files.zip › Supplementary_for_compiled PDF[2].pdf]

Figure S1

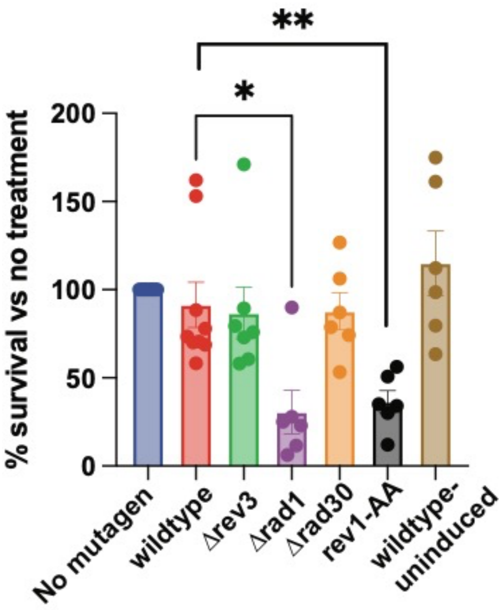

Fig S1: Viability of the strains from Fig 1 with acetaldehyde treatment, shown as percentage of survival compared to the corresponding control treated samples (100%). Data are shown as mean survival with error bars representing standard error of mean. Asterisks indicates statistical significance based on a p-value <0.05 from an unpaired t-test (wildtype vs Δ*rad1*= 0.01, wildtype vs *rev1-AA*= 0.005)

Figure S2

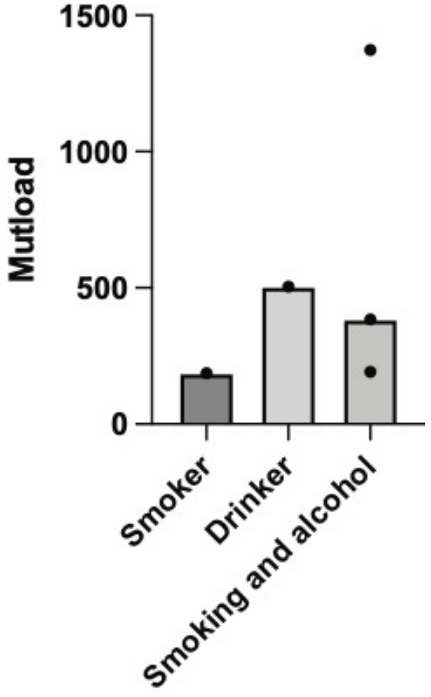

Fig S2: Clinical correlation of acetaldehyde mutation loads with smoking/alcohol status. Whole-genome sequenced esophageal carcinoma samples (ESCA) from PCAWG showing a statistically significant fold enrichment of the AA mutation signature were classified according to smoking/alcohol usage against gCn→gAn mutation loads within these samples. All samples with enrichment of the acetaldehyde signature were either smokers, drinkers, or both.

Supplementary table legends

Table S1: Yeast strains and oligonucleotide primers used in the study.

Table S2: Pilot data testing acetaldehyde mutagenesis in yeast ssDNA reporter system, along with source data for Fig 1 showing $Can^{R}Ade^{-}$ mutation frequencies in mutant yeast strains treated with AA or control.

Table S3: Phenotypic tests for whole-genome sequenced yeast strains treated with acetaldehyde or water.

Table S4: Source data for Fig 2. For samples ± acetaldehyde, single base substitutions, combined unique indels, mutation density per isolate, distribution of mutations in left and right sub-telomeric regions, distance of mutations from telomeres, and mutation spectrum per isolate for water vs AA-treated samples are shown.

Table S5: Source data for Fig 3A showing the number of mutations within all possible 96 trinucleotide contexts.

Table S6: Source data for Fig 3B showing the pentanucleotide sequence contexts for PLogo analysis.

Table S7: Source data for Fig 3C showing analysis of enrichment for the gCn→gAn mutation signature in whole genome sequenced yeast.

Table S8: Source data for Table 1 showing analysis of enrichment for the gCn→gAn mutation signature in whole exome sequenced ICGC cancers. Only cancers with samples having an enrichment of ≥1 (BH-corrected Fisher's p-value ≤0.05) are shown.

Table S9: Source data for Fig 4A showing correlation between the AA gCn→gAn signature and cumulative CC→AA (CC→AA + GG→TT) dinucleotide base substitutions in the indicated ICGC cancer cohorts.

Table S10: Source data for Fig Table 1 showing analysis of enrichment for the gCn→gAn mutation signature in whole genome sequenced PCAWG cancers. Only cancers with samples having an enrichment of ≥1 (BH-corrected Fisher's p-value ≤0.05) are shown.

Table S11: Source data for Fig 4B showing correlation between the AA gCn→gAn signature and cumulative CC→AA (CC→AA + GG→TT) dinucleotide base substitutions in the indicated PCAWG cancer cohorts.

Table S12: Source data for Table 1 for the analysis of enrichment for the gCn→gAn mutation signature in whole exome sequenced ICGC cancers for cancers not displaying an enrichment of ≥1 (BH-corrected Fisher's p-value ≤0.05).

Table S13: Source data for Table 1 for the analysis of enrichment for the gCn→gAn mutation signature in whole genome sequenced PCAWG cancers for cancers not displaying an enrichment of ≥1 (BH-corrected Fisher's p-value ≤0.05).

Table S14: Source data for Fig S2 showing the tobacco and alcohol exposure clinical data for whole-genome sequenced ESCA (esophageal carcinoma) samples from PCAWG.

Table S15: Source data for Fig 5A and 5B showing the transcriptional strand bias of the gCn→gAn acetaldehyde mutation signature in ICGC and PCAWG cancers displaying an enrichment of the AA mutation signature.
